# Three cases of retroperitoneal sarcoma in which bioabsorbable spacers (bioabsorbable polyglycolic acid spacers) were inserted prior to carbon ion radiotherapy

**DOI:** 10.1093/jrr/rrac002

**Published:** 2022-02-12

**Authors:** Itsuko Serizawa, Yohsuke Kusano, Kio Kano, Satoshi Shima, Keisuke Tsuchida, Yosuke Takakusagi, Nobutaka Mizoguchi, Tadashi Kamada, Daisaku Yoshida, Hiroyuki Katoh

**Affiliations:** Department of Radiation Oncology, Kanagawa Cancer Center, Kanagawa 241-8515, Japan; Section of Medical Physics and Engineering, Kanagawa Cancer Center, Yokohama 241-8515, Japan; Department of Radiation Oncology, Kanagawa Cancer Center, Kanagawa 241-8515, Japan; Department of Radiation Oncology, Kanagawa Cancer Center, Kanagawa 241-8515, Japan; Department of Radiation Oncology, Kanagawa Cancer Center, Kanagawa 241-8515, Japan; Department of Radiation Oncology, Kanagawa Cancer Center, Kanagawa 241-8515, Japan; Department of Radiation Oncology, Kanagawa Cancer Center, Kanagawa 241-8515, Japan; Department of Radiation Oncology, Kanagawa Cancer Center, Kanagawa 241-8515, Japan; Department of Radiation Oncology, Kanagawa Cancer Center, Kanagawa 241-8515, Japan; Department of Radiation Oncology, Kanagawa Cancer Center, Kanagawa 241-8515, Japan

**Keywords:** polyglycolic acid spacer, carbon ion radiotherapy (CIRT), retroperitoneal sarcoma

## Abstract

From August 2019 to August 2020, we inserted polyglycolic acid (PGA) spacers and administered carbon ion radiotherapy (CIRT) to three cases of retroperitoneal sarcoma at our hospital. We aimed to investigate its utility and safety for retroperitoneal sarcoma. We analyzed changes in PGA spacer volume and corresponding computed tomography (CT) values in addition to the dose distribution using in-room CT images that were obtained during treatment. We assessed adverse events and investigated the suitability, safety and effectivity of PGA spacer insertion. During treatment, changes in PGA spacer volumes and CT values were confirmed. Volumes increased in patients with a folded PGA spacer, and it increased 1.6-fold by the end of irradiation compared with planning CT. The CT values decreased by 20–50 Hounsfield units at the end of irradiation compared to the planning CT. Dose distribution evaluation showed that the dose to the gastrointestinal tract adjacent to the tumor was maintained below the tolerable dose, and a sufficient dose was delivered to the target by PGA spacer insertion. One case of subileus caused during abdominal surgery for PGA spacer insertion occurred. No other adverse events, such as digestive disorders, were observed. CIRT with PGA spacer insertion for retroperitoneal sarcomas is safe and effective. For cases in which there is no option but to perform irradiation using a PGA spacer, precautionary measures such as verification of dose distributions using CT images are necessary.

## INTRODUCTION

While surgical resection is the definitive treatment option for retroperitoneal sarcoma, there are many cases in which the anatomical characteristics make it difficult to resect with a negative margin. These tumors are adjacent to the viscera, and wide surgical resection with microscopically negative margins is usually not possible despite gross complete resection. Microscopically positive histologic margins also have a strong influence on both local control and overall survival [1–5]. Therefore, radiation therapy has been considered and tested as a preoperative and postoperative adjuvant therapy [6]. For cases of inoperable retroperitoneal sarcoma, the general options are radiation therapy with or without chemotherapy. Conventional radiation therapy using photon beams, such as X-ray, requires high dose irradiation to control sarcomas. However, because of the limited radiation tolerance of the retroperitoneal, abdominal and pelvic organs, adequate radiation doses are difficult to deliver. [7–9].

One physical characteristic of carbon ion beams is the Bragg peaks, which make it possible to concentrate the radiation dose deep in the trunk [10, 11]. In addition, they exhibit high linear energy transfer and exhibit high biological effects [12–15]. Carbon ion radiotherapy (CIRT) is proven to be effective, with treatment outcomes that compare well with surgery, even for pelvic sarcoma and chordoma, which are in the bone and soft tissue regions, difficult to resect and resistant to photon beams such as X-rays [16–18]. The superior physical and biological characteristics of carbon ion beams also yield satisfactory effects against retroperitoneal tumors [19].

However, when the tumor is adjacent to the digestive tract, there is a high risk of radiation enteritis, and hence the CIRT dose needs to be reduced near the digestive tract. To this end, methods of inserting a spacer between the tumor and digestive tract prior to performing carbon ion therapy have been tested [20, 21]. There are cases in which *in vivo* tissue is used as a spacer, along with cases in which an artificial object is used as a spacer. One of the disadvantages of an *in vivo* tissue spacer is that it is difficult to maintain the space as intended. Moreover, cases using artificial objects, such as GORE-TEX® (W. L. Gore & Associates, Inc., Newark, DE, USA), are at risk of infection and require surgical removal following the performance of CIRT [22, 23]. To solve these problems, a new bioabsorbable polyglycolic acid (PGA) spacer (Neskeep®, Alfresa Pharma Co., Osaka, Japan) has been developed [24]. These PGA spacers are 200 mm in height and 100 mm in depth, with three different thicknesses (5 mm, 10 mm and 15 mm). Following insertion into the body, they undergo hydrolysis and are absorbed. In a clinical trial, Sasaki *et al.* reported the safety and effectiveness of inserting PGA spacers prior to proton therapy. They also reported that, 8 weeks after the insertion of the PGA spacer and conclusion of proton therapy, 80% of the thickness at the time of insertion was maintained [25]. That is, when a PGA spacer with a thickness of 5 mm was inserted, a gap of 4 mm was retained following the conclusion of treatment.

In this study, we analyzed changes in volume and computed tomography (CT) values of the PGA spacer, dose distribution and adverse events using in-room CT images of retroperitoneal sarcoma treated with CIRT using the PGA spacer.

## MATERIALS AND METHODS

### Subjects

From August 2019 to August 2020, our facility identified 18 cases of retroperitoneal and pelvic sarcoma as suitable cases for CIRT, and five were considered suitable for the insertion of PGA spacers. At our hospital, for cases in which there was extensive contact between the tumor and digestive tract, and in which it was assumed impossible to administer the radiation dose necessary for radical treatment of the tumor, conferences were held by specialists in the Department of Musculoskeletal Tumor Surgery, Department of Diagnostic Radiology and Department of Radiation Oncology in order to consider the indication for spacer insertion. In one of the five cases, the tumor suddenly grew prior to CIRT, cropping out from the skin. In another case, adhesion was confirmed during surgery and damage to the digestive tract occurred; therefore, it was determined that the insertion of a PGA spacer would not be possible in those two cases. In the other three cases of retroperitoneal sarcoma in which PGA spacers were inserted prior to performing CIRT, changes in the PGA spacer volume and CT value, radiation dose distribution and adverse events were assessed.

### Bioabsorbable spacers

The volume was measured to investigate whether the thickness was maintained, so as to avoid bias at the measurement site. When PGA spacers undergo hydrolysis inside the body, carbon dioxide gas is created in the body, which may increase the thickness of the spacer or generate carbon dioxide gas or the stagnation of liquid between the PGA spacer if it is folded in half prior to insertion. Taking into consideration the role of the gap between the digestive tract and tumor, we measured the volume of the spacer while including carbon dioxide gas and the stagnation of the liquid that was produced. Furthermore, with regard to the CT values, we investigated changes using the median value of the PGA spacers. In principle, in-room CT was performed once a week, with additional imaging performed as necessary.

### Spacer insertion surgery

In all cases, PGA spacers were inserted between the tumor and digestive tract via ventrotomy. In one case (Case 3), the resectable left abdominal wall tumor and unresectable left retroperitoneal tumor were adjacent, so CIRT was conducted after simultaneously cutting the resectable lesion in the lumbar region and performing spacer insertion surgery.

The PGA spacers were 200-mm wide and 100-mm deep. For Case 1, one 1-cm-thick and one-quarter of a 5-mm-thick sheet were used. For Case 2, one and a half 1-cm-thick sheets were used and for Case 3, one 5-mm-thick sheet was used.

### CIRT

For the targets, healthy organs and PGA spacers, we used MIM maestro software version 5.6 (MIM Software Inc., Cleveland, OH, USA). To calculate dose distribution, we used Monaco version 5.20 carbon scanning system (Elekta AB, Stockholm, Sweden).

Gross tumor volume (GTV) was confirmed using CT images, MR images and PET-CT images. Clinical target volume (CTV) was standardized to a margin of 5 mm around the entire circumference of the GTV, excluding the area in which there was bone and the like with no infiltration and no conspicuous tumor development. A margin of 5 mm around the entire circumference of the CTV was secured for planning target volume (PTV). The administered radiation dose was 70.4 Gy RBE/16 fractions. Treatment planning established the objectives of GTV, CTV and PTV: V95% (volume irradiated with 95% of the treatment planning dose) as 95% and V90% (volume irradiated with 90% of the treatment planning dose) as 98%. The D2 cm^3^ (maximum dose that covered 2 cm^3^) of the digestive tract around the circumference of the tumor was also assessed.

All CIRT rooms at our hospital are equipped with in-room CT scanners as image-guided radiotherapy (IGRT), which were the same type as that used for treatment planning [26, 27]. CT could be performed with the patient set-up (2D-3D bone matching) maintained during treatment, thereby enabling appropriate in-room CT during the treatment period. In-room CT was performed for the first time for CIRT and weekly thereafter, with additional imaging when necessary.

The homogeneity index (HI) was calculated using the equation published in ICRU83 [28]. HI indicates the uniformity of the radiation fields.

$$ \mathrm{HI}=\left({\mathrm{D}}_{2\%}-{\mathrm{D}}_{98\%}\right)/{\mathrm{D}}_{50\%} $$

where ${\mathrm{D}}_{2\%}$ indicates the maximum dose, ${\mathrm{D}}_{98\%}$ indicates the minimum dose and ${\mathrm{D}}_{50\%}$ indicates the mean of the maximum and minimum doses.

**Table 1:** Characteristics of patients

	Age	Gender	Location	Adjacent digestive tract	Histopathology	Tumor length (cm)	Tumor volume (cm^3^)	History of surgery	History of chemotherapy	History of radiotherapy
Case 1	61	Men	Left kidney (infiltration)-left iliopsoas muscle-second lumbar vertebra	Intestinum duodenum Sigmoidal colon	Dedifferentiated liposarcoma	12.9	788.2	No	Yes	No
Case 2	67	Men	Right kidney-iliopsoas muscle-iliac fossa	Intestinum duodenum Ascending colon Intestinum caecum	Dedifferentiated liposarcoma	13.5	1006.1	No	Yes	No
Case 3	73	Men	Extremitas inferior of the left kidney-left iliopsoas muscle	Descending colon	Dedifferentiated liposarcoma	1.8^*^	8.1^*^	Yes	Yes	Yes (photon)

^*^Lesion subject to CIRT, excluding the tumor that had been resected

## RESULTS

The characteristics and treatment of cases are indicated in Table 1. The performance status of all the patients was 0. The ages of the patients were 61, 67 and 73 years. All cases involved retroperitoneal sarcomas. In Case 1, a tumor was confirmed from the left kidney-left iliopsoas muscle-second lumbar vertebra, and infiltration to the kidneys and the second lumbar vertebra was confirmed. The adjacent digestive tract was the intestinal duodenum and sigmoidal colon. In Case 2, a tumor was confirmed from the dorsal side of the right kidney-iliopsoas muscle-iliac fossa, while the adjacent digestive tract was the intestinum duodenum and ascending colon. In Case 3, the lesion subject to CIRT was confirmed from the extremities inferior to the left kidney-left iliopsoas muscle, while the adjacent digestive tract was the descending colon.

The histopathology in three cases was dedifferentiated liposarcoma. All three patients had a history of chemotherapy, and one patient had a history of surgery and X-ray irradiation. The X-ray and CIRT fields were overlapped and the administered dose of X-rays was 60 Gy/30 fractions/43 days. In all cases, PGA spacers were inserted using ventrotomy with anti-adhesion agents used to prevent adhesion.

The follow-up periods were 24, 2 and 19 months after the start of CIRT. No local recurrence was observed during the observation period.

In Case 1, subileus occurred following insertion surgery; therefore, inanition management was adopted. The CIRT was pushed back to 1 week behind the schedule. There were no complications following insertion surgery in the other two cases.

In Case 2, the pain caused by the tumor made it impossible to maintain a posture with the knees extended, so it was impossible to create a fixture in the abdominal position as planned. Therefore, a fixture was created in the dorsal position with the knees bent. As the carbon ion radiography machines at our hospital were not fitted with a gantry, irradiation was performed from the abdominal side, as it was impossible to do so from the dorsal side. Therefore, the vertical beam passed through the PGA spacers.

**Fig. 1.**
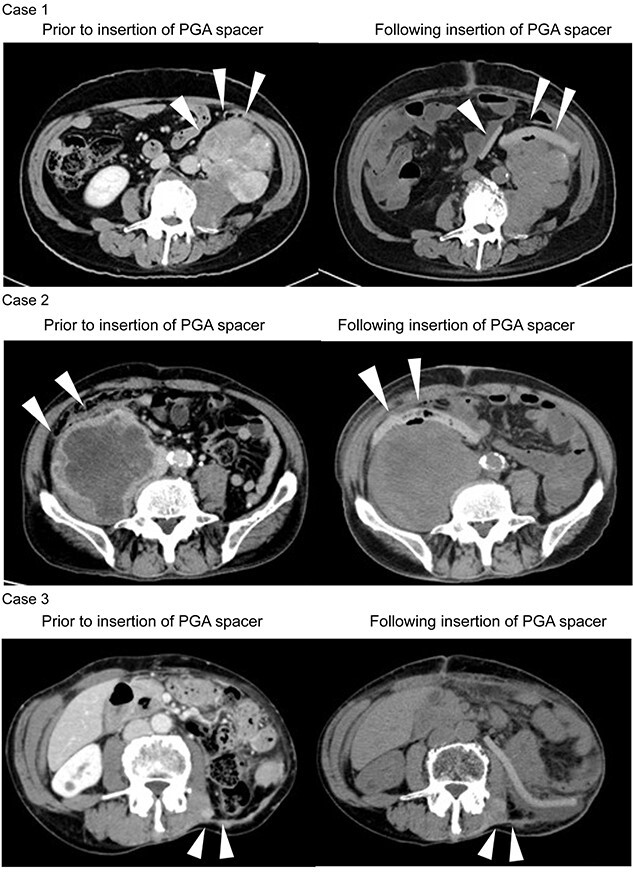
CT images before and after insertion of PGA spacer. Arrows: Location where there is contact between the tumor and the digestive tract.

**Fig. 2.**
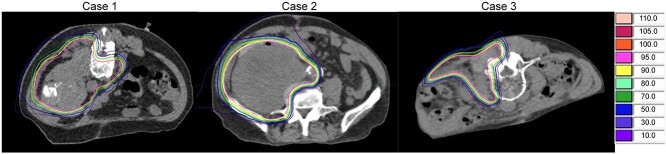
Diagram of radiation dose distributions in each case.

Fig. 1 shows the CT images before and after insertion in each case. Fig. 2 shows the dose distribution in the treatment plan. Table 2 shows the changes in the distance between the tumor and digestive tract at the position with the greatest contact between the tumor and digestive tract for each case. Table 3 shows the dosimetric parameters in the initial treatment plan.

The areas with decreased radiation dose to the targets in Cases 1 and 3 experienced infiltration of the tumor into the vertebral body and contact with the skin, requiring a reduction in the radiation dose to the inside of the vertebral canal and the skin. In each case, PGA spacers were able to maintain the distance between the tumor and digestive tract, thereby minimizing the radiation dose administered to the digestive tract.

### PGA spacer

**Fig. 3.**
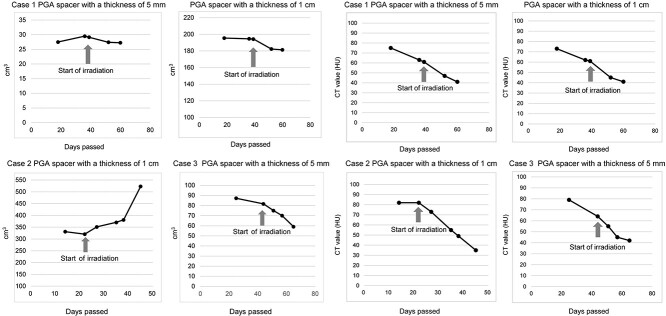
(a) Changes in volume of PGA spacers. (b) Change in CT values of bioabsorbable spacers.

**Fig. 4.**
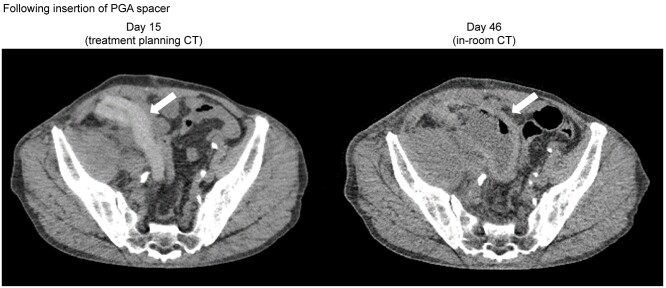
The treatment planning CT image and in-room CT image (46 days after the insertion of PGA spacer) of Case 2. Arrow: PGA spacer.

Fig. 3 shows the changes in volume and CT values of the PGA spacers. In Case 2, carbon dioxide gas and liquid stagnation were confirmed in the gap in which the PGA spacer was folded in half, and expansion of the volume was confirmed. Fig. 4 shows the treatment planning CT image and in-room CT image (46 days after the insertion of the PGA spacer). A reduction in coverage was observed; therefore, irradiation was performed under a new treatment plan from the 28th day following insertion surgery. The coverage and HI of the perpendicular beams in the treatment plan prior to change (PLAN 1) and following change (PLAN 2) of Case 2 are shown in Table 4.

In Case 3, a volume of 80% was maintained 8 weeks following insertion surgery, but it decreased to 68% at the 13th irradiation.

With regard to CT values, there was a 20 to 50 HU decrease from the time of CT for the treatment plan to the end of irradiation.

**Table 2:** Changes in distance between the tumor and digestive tract (at the position with the greatest contact between the tumor and digestive tract)

	Prior to insertion of PGA spacer (mm)	Following insertion of PGA spacer (mm)
Case 1	0	10
Case 2	0	10
Case 3	3	19

PGA, polyglycolic acid

### Adverse events accompanying CIRT and PGA spacer

Subileus following insertion surgery was observed in one patient, but no other grade 2 or higher acute adverse events were observed during the follow-up period. Hydronephrosis associated with ureteral stenosis due to CIRT was observed in one patient at 7 months after CIRT.

## DISCUSSION

Soft tissue sarcoma (STS) is a rare type of tumor that accounts for only 1% of all malignant tumors. Retroperitoneal sarcoma is an extremely rare type of tumor, accounting for 10% of STS cases, and affects only 0.5 to 1 person per 100 000 persons every year [29, 30]. Regarding histopathology, liposarcoma and leiomyosarcoma account for approximately 60% of cases [31, 32]. Retroperitoneal sarcomas have a high rate of local recurrence. Since 75% of cancer-related deaths caused by retroperitoneal sarcoma are due to the inability to control the cancer locally, local control is required for prognostic improvement [33].

CIRT yields a high dose concentration as well as biological effects, so irradiation can be performed for radical treatment of inoperable STS, and satisfactory treatment outcomes have been reported [16–19]. However, there are concerns about damage to the digestive tract in tumors that are adjacent to the digestive tract, so it is difficult to administer the radiation dose for radical treatment of the tumor. To this end, spacers are fitted between the tumor and digestive tract.

**Table 3:** Dosimetric parameters in target and organs at risk (at initial plan)

Organs	Criteria	Judgment criteria	Patient 1 (%)	Patient 2 (%)	Patient 3 (%)
GTV	V95%	95%	96.54	99.99	91.8
	V90%	98%	97.46	100	99.85
CTV	V95%	95%	94.64	99.55	87.48
	V90%	98%	95.96	99.92	93.93
PTV	V95%	95%	90.22	96.8	88.5
	V90%	98%	93.72	99.74	99.24
Bowel	D2cc	40Gy RBE	36.72	34.86	28.46

GTV: gross tumor volume CTV: clinical target volume PTV: planning target volume.

Bowel: Digestive tract surrounding the tumor

**Table 4:** GTV coverage and HI of horizontal beams

Days since PGA spacer insertion	Day 15	Day 23	Day 28	Day 36	Day 39	Day 46
GTV volume (cm^3^)	1001.1	1219.1	1262.9	1239.4	1237.9	1248.4
PLAN 1						
GTV V95%	99.98	96.8	97.72	97.29	97.1	96.97
HI	0.023	0.097	0.07	0.081	0.084	0.088
PLAN 2						
GTV V95%	100	99.94	100	99.98	99.94	99.98
HI	0.016	0.022	0.02	0.022	0.022	0.022

PLAN 1: Initial treatment planning, PLAN 2: Treatment planning following change.

PGA, polyglycolic acid; GTV, gross tumor volume; HI, homogeneity index; RBE, relative biological effectiveness

Regarding the indication for fitting spacers, this may be unsuitable for cases in which the tumor has infiltrated the digestive tract and in cases in which adhesion with the digestive tract would make it difficult to insert the spacer. Moreover, one disadvantage is that the insertion of a spacer requires surgery, which would delay the start of treatment compared to cases without insertion of a spacer. At our hospital, approximately 5 weeks is required to make a decision on the suitability of a spacer to the start of irradiation, which means that irradiation is initiated approximately 3 weeks later than for cases in which there is no insertion. Since a delay in treatment can be fatal in cases of rapidly growing tumors, cases of rapid tumor growth are unsuitable for insertion. Therefore, to determine the suitability of inserting a spacer, the rate of growth of the tumor must be considered.

In our cases, 80% of the PGA spacer volume was maintained 8 weeks later. There was one case where the volume had decreased to 68% by the 10th week of irradiation. In *in vivo* experiments on mice, it has been reported that PGA spacers can be maintained with a stable thickness of 90% up until 8 weeks following insertion, subsequently decreasing after 12 weeks [25]. PGA spacers are considered ideal for cases in which CIRT is conducted within 8 weeks following insertion of the spacer. A PGA spacer inserted with folds showed gas and liquid inside and a significant increase in volume, and this possibly indicated an increase in body thickness change and infection risk; therefore, further study on the insertion with two folds is considered necessary.

At our hospital, appropriate in-room CT is performed during treatment. This enabled us to recognize the changes in the PGA spacer, tumor size and changes in dose coverage during irradiation. Therefore, in Case 2, it was possible to recognize a decrease in coverage and change the plan. A comparison of the horizontal beams from the treatment plan prior to the change (PLAN 1) to the treatment plan following the change (PLAN 2) indicated a decline in coverage and worsening of the uniformity of the radiation dose for cases in which treatment was continued under PLAN 1, while no problems were confirmed with regard to the coverage and uniformity of the dose in PLAN 2. In scanning CIRT, the irradiation position is skillfully determined and irradiated spot by spot. The presence of an air layer is more likely to affect the beam range compared to the conventional passive method. Therefore, we evaluated not only the coverage but also the uniformity of the radiation dose.

Using imaging in-room CT, changes during treatment could be recognized, thus preventing dose administration reduction. The changes in coverage related to the PGA spacer were considered as follows: changes in the thickness of the PGA spacer, internal gas, liquid storage in the gap when folded in two, and changes in the CT value of the PGA spacer. In this case, it was thought that the stagnation of gas in the PGA spacer and a decrease in the CT value shrunk the range, and the expansion of the thickness due to the stagnation of liquid in the gap extended the range. The causes of the change in coverage related to the patient were considered to be expansion of the body contour and growth of the tumor. Because there is a complicated mixture of multiple factors, cases in which irradiation through the PGA spacer is unavoidable, due to reasons such as difficulty in maintaining the position of the body, as in this case, appropriate CT and assessment of the radiation dose distribution are required.

One adverse event, subileus, occurred in one patient as a result of abdominal surgery for insertion of the PGA spacer and this delayed CIRT by 1 week. Lorenzo *et al*. reported the insertion of a silicon sheet spacer using a laparotomic or hand-assisted laparoscopic approach [34]. PGA spacers are inserted using ventrotomy; however, future consideration of endoscopic insertion may prevent factors such as delays in treatment. This study did not confirm any other case of acute phase damage to the digestive tract as a result of CIRT.

Hydronephrosis due to ureteral stenosis on the affected side was observed as a late adverse event of CIRT. The stenosis part of the uterus was in the irradiation field and was irradiated at the full dose. If it was possible to have inserted the spacer so that the ureter was on the gastrointestinal side rather than the tumor side during PGA spacer insertion, the ureter would have been out of the irradiation field, which may have prevented the damage. When inserting a spacer for a retroperitoneal sarcoma, the radiation oncologist should discuss the ureter with the surgeon.

While no late-stage damage to the digestive tract was confirmed, it is important to remember that the observation period in this study was short at less than 2 years, it is necessary to carefully confirm the longer term outcomes of this process.

In conclusion, by PGA spacer insertion dose distribution evaluation showed that the dose to the gastrointestinal tract adjacent to the tumor was maintained below the tolerable dose, and a sufficient dose was delivered to the target. PGA spacers are effective in CIRT against retroperitoneal sarcomas that are adjacent to the digestive tract, with early-stage adverse events within the permissible range.
